# Exosomes: From Potential Culprits to New Therapeutic Promise in the Setting of Cardiac Fibrosis

**DOI:** 10.3390/cells9030592

**Published:** 2020-03-02

**Authors:** Roman Tikhomirov, Benedict Reilly-O’Donnell, Francesco Catapano, Giuseppe Faggian, Julia Gorelik, Fabio Martelli, Costanza Emanueli

**Affiliations:** 1 National Heart and Lung Institute, Imperial College London, Hammersmith Campus, Du Cane Road, London W12 0NN, UK; roman.tikhomirov19@imperial.ac.uk (R.T.); b.reilly-odonnell@imperial.ac.uk (B.R.-O.); f.catapano@imperial.ac.uk (F.C.); j.gorelik@imperial.ac.uk (J.G.); 2 Department of Surgery, Dentistry, Pediatrics and Gynecology, Cardiovascular Science, The University of Verona, Policlinico G., B. Rossi, P.le. La Scuro 10, 37134 Verona, Italy; giuseppe.faggian@univr.it (G.F.); Fabio.martelli@grupposandonato.it (F.M.); 3 Molecular Cardiology Laboratory, IRCCS Policlinico San Donato, Via Morandi 30, 20097 San Donato Milanese Milano, Italy

**Keywords:** cardiac fibrosis, heart failure, extracellular vesicle (EVs), EVs engineering, exosomes, microRNAs, noncoding RNAs, stem cells

## Abstract

Fibrosis is a significant global health problem associated with many inflammatory and degenerative diseases affecting multiple organs, individually or simultaneously. Fibrosis develops when extracellular matrix (ECM) remodeling becomes excessive or uncontrolled and is associated with nearly all forms of heart disease. Cardiac fibroblasts and myofibroblasts are the main effectors of ECM deposition and scar formation. The heart is a complex multicellular organ, where the various resident cell types communicate between themselves and with cells of the blood and immune systems. Exosomes, which are small extracellular vesicles, (EVs), contribute to cell-to-cell communication and their pathophysiological relevance and therapeutic potential is emerging. Here, we will critically review the role of endogenous exosomes as possible fibrosis mediators and discuss the possibility of using stem cell-derived and/or engineered exosomes as anti-fibrotic agents.

## 1. Introduction

### 1.1. Introduction: Fibrosis

Fibrosis is a well-recognized cause of morbidity and mortality. Fibrotic diseases cause more than 800,000 deaths worldwide annually, the majority of which are due to lung and cardiac complications [1]. Cardiac fibrosis is a process of pathological extracellular matrix (ECM) remodeling which affects the quality and quantity of the ECM of matrix composition, over time this can impair the heart physically and electrically, vastly reducing cardiac function. Cardiac fibrosis is dominant in myocardial infarction (MI)-induced heart failure (HF) with reduced ejection fraction (HF-rEF), but it accompanies almost every form of cardiac disease, such as hypertensive heart disease, diabetic cardiomyopathy and idiopathic dilated cardiomyopathy. Moreover, fibrosis can also be induced by therapeutic interventions, such as radiation therapy. Cardiac fibrosis induces pathological changes that increase myocardial stiffness, cardiomyocyte hypertrophy, and ventricle chamber dilatation, ultimately leading to the development of congestive HF. Indeed, the level of cardiac fibrosis can be used as a predictor of adverse outcomes in HF patients [2,3]. There are several different types of cardiac scars depending upon location and the underlying cause (reviewed in [4,5]). In this article, we focus principally on two types of cardiac fibrosis, which are the most relevant for the remodeling of the ischemic adult heart: (1) reactive interstitial fibrosis is characterized by an increase in collagen synthesis and diffused deposition of collagen that leads to an increased interstitial compartment volume without loss of myocytes. This type of fibrosis occurs progressively in response to increased pressure and/or volume loads as in the cases of hypertension, aortic stenosis, ageing, and diabetes. Reactive interstitial fibrosis is potentially reversible through curtailing the damaging stimuli or by targeted therapies. (2) Diffuse or focal replacement fibrosis follows cardiomyocyte death, typically after a MI. In replacement fibrosis, which is currently not reversible, the affected myocardium is not viable and thus unable to recover contractile properties.

#### 1.1.1. Cardiac Fibroblasts

Under homeostatic conditions, the fibroblast-produced ECM provides a structural scaffold for cardiomyocytes, distributes mechanical forces through the cardiac tissue, and mediates electric conduction. The post-natal mammalian heart has very limited regenerative capacity after injury. Following an MI, cardiomyocyte necrosis triggers an inflammatory phase guided by neutrophils, which leads to activation of cardiac fibroblasts to become myofibroblasts. The myofibroblasts then form a scar, acting to preserve structural and functional integrity of the myocardium. Resident cardiac fibroblasts are the main cell type contributing to cardiac fibrosis, but their identity, functional properties, and activation dynamics are still poorly understood [6].

#### 1.1.2. Molecular Mechanisms of Cardiac Fibrosis

Pathological remodeling of the myocardium, at a cellular level, commences with changes in cellular behavior. Effector cells like fibroblasts and pericytes can transdifferentiate into myofibroblasts [7]. There are various mechanisms which stimulate this process including mechanical and chemical signals [8]. Myofibroblasts produce alpha smooth muscle actin (α-SMA) and myosin, which form connections with focal adhesion proteins, binding cellular actin filaments with the ECM. Mechanical stress can provoke further expression of α-, β-, and γ-fibers, connected with focal adhesion proteins [9]. In addition, myofibroblasts are factories of ECM protein production, particularly collagens which can cross-link and therefore become highly resistant to degradation by proteases [10].

**Figure 1:**
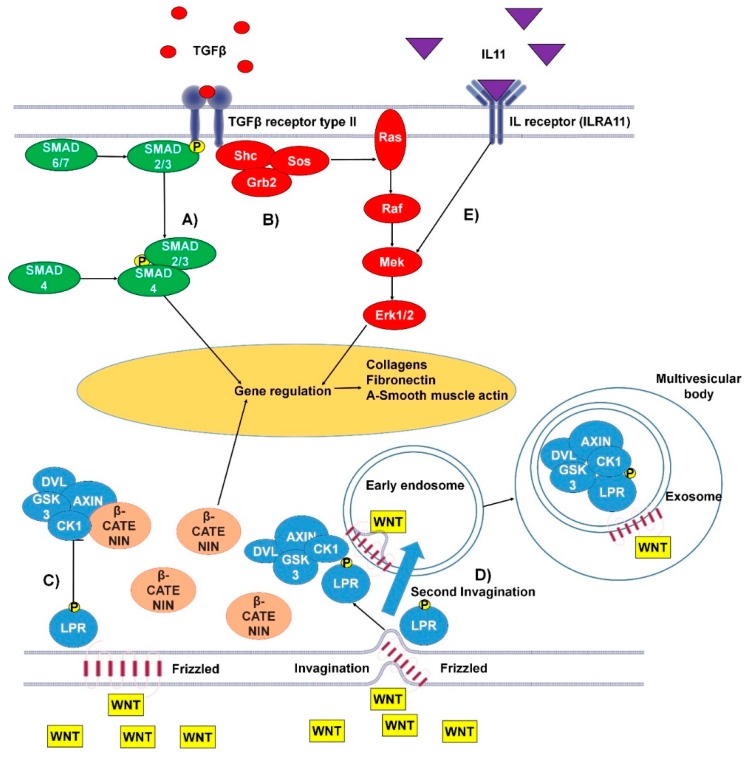
The canonical and non-canonical pro-fibrotic pathways of transforming growth factor β (TGFβ) and Wnt and the pro-fibrotic interleukin 11 (IL-11) pathway. (**A**) Canonical TGFβ pathway: TGFβ binds to the type I/II TGFβ receptor. The Smad2/3 complex is then phosphorylated, at this point the pathway can be inhibited by Smad6/7. In the cytoplasm Smad2/3 binds Smad4 and the whole complex transfers to the nucleus, where it binds to GAGAC motifs, promoting gene expression. (**B**) Non-canonical TGFβ pathway: TGFβ binds the TGFβ receptor type I/II, resulting in tyrosine residues and Shc (Src homology 2 domain containing) transforming protein phosphorylation. This promotes the binding of Grb2 (Growth factor receptor-bound protein 2) and Sos (son of sevenless). This complex can activate Ras, launching the MAPK cascade and further gene regulation. (**C**) Wnt canonical pathway: Wnt (wingless-related integration site) binds transmembrane protein frizzled (Fz). Fz bound WNT can then bind LPR5/6 protein (low-density-lipoprotein-related protein) which undergoes phosphorylation of its tail by GSK3 and CK1 proteins. Following this, low-density-lipoprotein receptor-related protein (LRP) interacts with Disheveld (DVL), Axin, and GSK3 via Pro-Pro-Pro-(Ser/Tyr)-Pro repeats. This complex is responsible for β-catenin attenuation. (**D**) Wnt non-canonical pathway: Wnt binds transmembrane protein Fz at the site of a membrane invagination. The complex then becomes part of the membrane of an early endosome. LPR5/6 is phosphorylated at its tail by GSK3 and CK1 proteins. Following this, LRP interacts with DVL, Axin, and GSK3 via Pro-Pro-Pro-(Ser/Tyr)-Pro repeats. The membrane of the early endosome can form a second invagination, eventually leading to the whole complex being locked down inside a multivesicular body. (**E**) The IL-11 signaling pathway activates the MAPK cascade, promoting gene regulation [15,16,24].

Several molecular mechanisms regulate cardiac fibrosis. In this review, we focus at those pathways, which are connected with exosomes biology. A summary of these mechanisms can be viewed in Figure 1.

#### 1.1.3. TGFβ Canonical and Non-Canonical Pathways

Transforming growth factor β (TGFβ) is a multifunctional cytokine, which is considered a main driver of cardiac fibrosis. Under pathological conditions, TGFβ is released from the ECM, where it is stored in complex with latency-associated peptides (LAPs) and latent TGFβ binding proteins [11]. There are two membrane-bound TGFβ receptors (types I and II), when activated by TGFβ the receptors activate the Smad pathway [12]. In the cytoplasm, phosphorylated Smad2/3 forms a heteromeric complex with Smad4; following this, the complex associates with DNA-binding proteins and is transported to the nucleus where GAGAC motifs are bound to initiate extensive expression of target genes. Such a cascade can be interrupted at the step of Smad2/3 phosphorylation due to inhibition by Smad6/7. Inhibition of the TGFβ- signaling pathways does not completely attenuate the progression of fibrosis, indicating that other pathways are also involved. Evidence for non-canonical pathways of TGFβ-signaling include activation of the MAPK pathway as demonstrated in work of Lu Xie et al. [13,14]. Briefly, this proposed pathway begins with the phosphorylation and activation of TGFβ binding receptors type I and II, which consequently activates Shc. Shc forms a complex with Grb2 and son of sevenless (Sos) protein. Consequently, the complex activates membrane embedded Ras leading to step by step activation of Raf, Mek, and Erk with a further involvement of MAPK cascades. As a result, Erk and Smad signaling influence expression of genes associated with fibrosis [15].

#### 1.1.4. IL-11 Signaling Pathway

Interleukin 11 (IL11) is a cytokine which was found to be upregulated in response of TGFβ1 stimulation of cardiac fibroblasts. The laboratory of Stuart Cook showed that IL11 binds interleukin receptor (ILRA11) and acts through ERK to regulate gene expression, in order to produce ECM proteins [16]. This group also showed that activation of the SMAD cascade through TGFβ1 results in high release of IL11 cytokine [16] and that IL11 stimulates lung fibroblasts and leads to generation of α-smooth muscle actin and collagens in an ERK-dependent post-transcriptional manner [17]. Similar results were observed in liver fibrosis in the model of nonalcoholic steatohepatitis mouse [18]. Therefore, the IL11 pathway appears to be involved in different models of fibrosis.

#### 1.1.5. Angiotensin II and Nuclear Factor-κβ

The fibrotic response of cardiac fibroblasts is not limited to TGFβ, other growth factors and cytokines can also stimulate this process. Pro-fibrotic cytokines are mostly released by cells involved in inflammation, such as macrophages and neutrophils. Angiotensin II and hypoxic conditions activate activator protein AP-1, which causes an increase in the expression of collagens, fibronectin, and intercellular cell adhesion molecule 1 (ICAM1) [19,20,21,22]. Moreover, it was also reported that exosomes from cardiac cells submitted to mechanical stretch to mimic HF can transport AT1 receptors. Nuclear factor-κβ (NF-κβ) is a transcription factor which is well known for its contribution in cardiac remodeling, heart failure, and hypertrophy [23].

#### 1.1.6. Wnt Pathways

Nowadays, the Wnt pathway is extensively studied. This has led to the identification of canonical and non-canonical Wnt-β-catenin pathways [24]. The Wnt pathways have been identified as participating strongly in the development of cardiac fibrosis [25]. Wnt3a causes upregulation of both TGFβ and Smad2 and induces proliferation of cultured mouse fibroblasts [26]. Additional connections between the Wnt canonical pathway and TGFβ pathway have been confirmed in other investigations [27,28]. The canonical Wnt pathway works through negative regulation of the complex which binds and inhibits β-catenin (which was found to be important in the promotion of fibrosis). The loss of function of β-catenin was reported to reduce interstitial fibrosis suppressing Col3a1 [29]. Signaling begins when Wnt binds to the transmembrane receptor Frizzled (Fz). Afterwards the receptor can interact with a low-density-lipoprotein-related protein (LRP5/6) with further phosphorylation of LRP tail by GSK3 and CK1 proteins. Following this, LRP interacts with Disheveld (DVL), Axin, and GSK3 via Pro-Pro-Pro-(Ser/Tyr)-Pro repeats. This complex is responsible for β-catenin attenuation through interaction with phosphorylated LRP. As a result, β-catenin translocates into the nucleus where, in a complex with T-cell factor/lymphoid enhancer-binding factor-1 (TCF/Lef-1), transcription factors and co-factors, it can regulate gene transcription [30]. Wnt is also closely connected to the biogenesis of extracellular vesicles (explained below in Section 1.2.1). Following binding between Wnt and Frizzled (transmembrane receptor), a membrane invagination occurs and early endosome forms. During the maturation of endosome, the MVB formation leads to trapping of the β-catenin inhibiting complex inside MVB. This complex can be released in the extracellular space with the exosomes [31].

### 1.2. An Introduction to Extracellular Vesicles

The adult human heart is made up of billions of cells, approximately a third of these (by number) are cardiomyocytes whilst the remaining 60%–70% are endothelial cells, fibroblasts, neural cells, and other vascular cell types [32]. The orchestrated function of different cardiac cell populations and patrolling immune cell subsets provide a complex network of intercellular circuits of communication, which are essential to cardiac homeostasis and repair [33,34]. In this context, extracellular vesicle (EVs) play fundamental roles. The term EVs denotes a highly diverse family of membrane vesicles of different biogenesis and sizes: apoptotic bodies (usually from 0.8 to 5 μm in diameter), microvesicles (from 0.1 to 1 μm) and exosomes (from 30 to 150 nm) [34,35]. This review will focus on exosomes. Exosomes are generated from the late endosomal pathway and actively secreted [36]. They contain endosomal membrane markers, such as tetraspanins, plus a composite molecular cargo that can include RNAs and proteins [37,38,39]. Exosomes are utilized by cardiac cells to communicate with infiltrating blood, immune cells, and between each other [36,40,41,42]. Exosomes protect their molecular cargo from degradation until it is passed on, in a functionally active status, to neighboring cells through binding, fusion, or endocytosis. Exosomes are incredibly diverse and can differ substantially in their cargo and membrane composition [43,44,45]. Consequently, the pool of exosomes that populate the extracellular space of the heart is both diverse and variable. The coordination of exosome-mediated communication is important for maintaining physiology, but if altered, it can support disease propagation [46,47]. This dual function can be explained by considering that the amount and quality of exosomes secreted from a cell is influenced by both its activation status and the external environment to which the cell is exposed [48,49], including hypoxia [50].

#### 1.2.1. Exosomes Biogenesis

**Figure 2:**
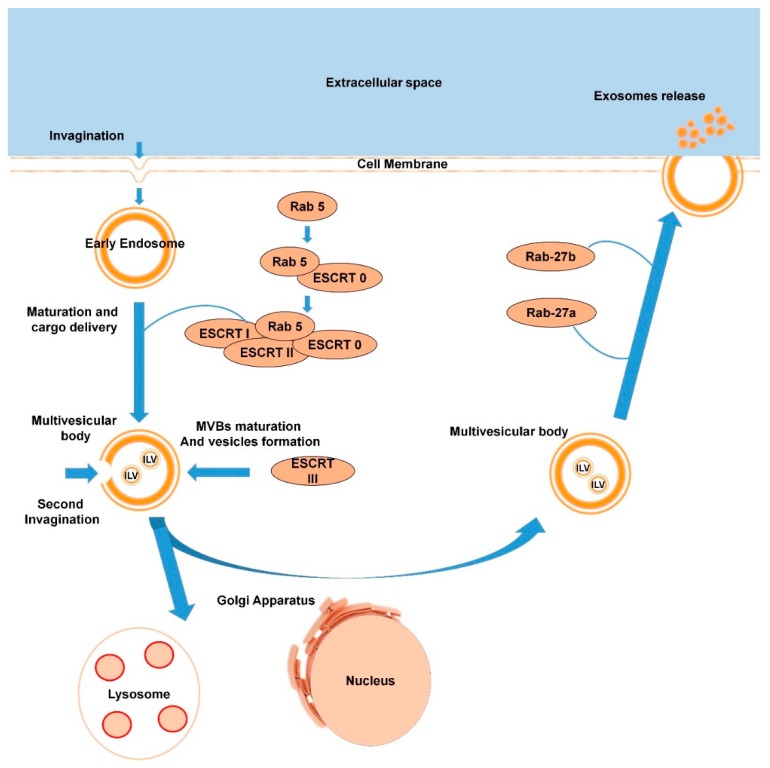
Scheme of exosome biogenesis (the process is described in detail in Section 1.2.1.).

Exosomes have a different release pathway compared to other EVs, which can influence their functional properties. Studying the mechanisms which underlie exosome maturation and release is therefore important and could be key to some therapeutic approaches [51]. As already mentioned in Section 1.1.6, exosomes are generated in MVBs matured from early endosomes. The process of biogenesis of exosomes begins with invagination of the cell membrane and formation of early endosomes in the cytosol. Several studies have shown that Rab-GTPases play a central role in the early endosome maturation and overall exosome formation since these proteins regulate vesicular traffic of membrane components and lysosomal hydrolases [52,53,54,55]. As example, Rab5 in its active form provides a platform for proteins with FYVE domain recognition and plays an important role in delivering cargo to the early endosomes [56]. It leads to further binding with a protein complex ESCRT-0, followed by finalization of the complex ESCRT-I and ESCRT-II. The next step is a second invagination of the matured endosomes, which leads to the formation of the intraluminal vesicles (ILVs) and ESCRT-III complex. This complex is responsible for multivesicular body (MVBs) formation [57]. During MVB maturation there are two destinations of future “exosomes”: they can be degraded by lysosomes (or Golgi apparatus), or they can be released into the extracellular space via MVBs fusion with the plasmalemma and become “real exosomes”. Rab27a and Rab27b play an important but not crucial role in the destiny of ILVs. Ostrowski and colleagues identified that inactivation of these proteins decreases exosome secretion by 50% [58]. Consequently, it is logical to assume that there are alternative mechanisms and additional factors influencing exosome release. Markus Babst, in his review, suggested a lipid-driven mechanism of ILV formation [59]. Catarina and colleagues report that ceramide is very important for exosome genesis [60]. Nevertheless, the authors admit to the fact that proving any model of ILV formation would require the development of new tools to study membrane-based systems. Once the exosomes are released into the extracellular space, they can be taken up by other cells or move around the body in the circulatory system. Exosome biogenesis is schematized in Figure 2.

#### 1.2.2. Exosome Uptake Mechanism

The mechanisms underlying exosome uptake by recipient cells remain a debatable and insufficiently studied topic. There are three suggested mechanisms of uptake: endocytosis, membrane fusion, and receptor-ligand mediated interactions [61,62]. Endocytosis itself has a variety of mechanisms such as phagocytosis, micropinocytosis, clathrin- and caveolae-mediated and independent endocytosis [63]. Tian Tian et al. revealed that exosomes derived from PC12 cells can be taken up by clathrin-mediated endocytosis and micropinocytosis [64]. However, recent work of Horibe and colleagues displayed that mechanism of exosomes uptake differed depending on the type of recipient cells [65]. Receptor-ligand binding and recent works on this topic were described clearly in a recent review [62].

Despite the unclear mechanism of exosome uptake, one fact remains obvious: exosomes can transfer biologically active molecules from parent cells to recipient cells and there is compelling evidence of this process occurring in a variety of cell types and diseases including cardiovascular diseases [66,67,68,69]. Interestingly, exosomes and their cargo are different in composition from their parent cells. This indicates that there is selective loading of EVs with functional molecules [70,71,72], the mechanisms for achieving this are described in the next section.

#### 1.2.3. Mechanism of ncRNA and Protein Cargo Loading into Exosomes

Exosomes can embed very different molecules from parent cells including proteins, non-coding RNAs (ncRNAs), lipids, mRNAs, and even small DNA fragments [73,74]. Profiling studies support the idea that miRNAs, lncRNAs, and circRNAs are loaded following the instruction of regulatory systems. Mechanisms of loading for miRNAs were reviewed by Zhang and colleagues and summarized as four main pathways and one other potential mechanism [75]. (1) The neural sphingomyelinase 2 dependent pathway [76], (2) heterogeneous nuclear ribonucleoprotein-dependent pathway, which includes hnRNPA2B1 [77], (3) the 3′-end of the miRNA sequence dependent pathway [78], and (4) the miRNA induced silencing complex-related pathway [79]. Recently it was shown that hnRNPA2B1 can be also important in loading of lncRNAs inside exosomes [80]. Recent work of Diana Cha and colleagues has identified another example of selective loading of EVs with miRNAs and lncRNAs. Their investigation compared exosomal RNAs from colorectal cancer cells, with and without a mutant KRAS [70,71,72,73,74,75,76,77,78,79,80,81]. As a result of this study, it was found that miRNAs, lncRNAs, and mRNAs are loaded in a selective manner and that these molecules can be transferred from cell to cell via exosomes. Furthermore, it was suggested that exosomes from the KRAS-mutant cells could induce the disease phenotype in non-mutant cells. Importantly, there was a global reduction of circRNAs in the mutant KRAS cells, which was paralleled by increased circRNA in their EVs [71].

Proteins are usually loaded into exosomes with the help of the ESCRT-complex, as described in the previous section [82]. However, inhibition of ESCRT-complex activity does not completely attenuate exosome genesis and sorting [83]. Vidal M., et al. showed that some molecules were released in extracellular space via their association with lipid raft domains of exosomal membrane [84].

In summary, the molecular cargo loaded into exosomes is not random, and this fact suggests there are opportunities to therapeutically modify the cargo of exosomes as a strategy for disease treatment. Fundamental studies on the mechanisms of fibrosis will help to progress our understanding of ncRNAs and other molecules able to attenuate the disease. Alternatively, antisense sequences and inhibitors can be loaded in exosomes to silence pro-fibrotic molecules in recipient cells. This review will focus on exosomes for treatment of cardiac fibrosis. As a consequence, exosome cargo components that can modulate fibrosis will be discussed in subsequent sections.

## 2. Exosomes for the Treatment of Cardiac Fibrosis

**Figure 3:**
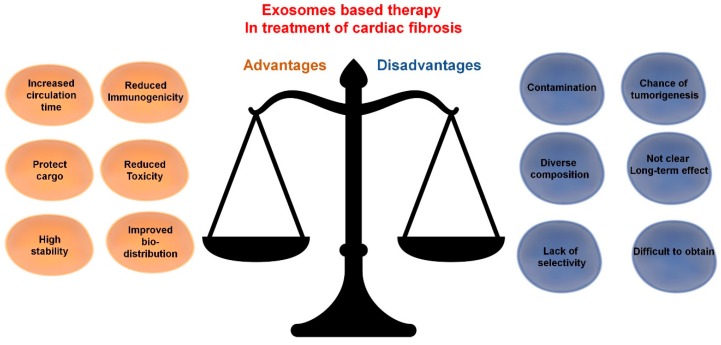
Exosome-based therapy in treatment of cardiac fibrosis: advantages and disadvantages.

When compared to conventional drugs, exosome-based therapies could improve cellular and tissue distribution of the curative bioactive molecules and hence improve efficacy and reduce toxicity. Moreover, in comparison to stem cell treatments, exosomes could additionally reduce immunogenicity. Inspired by these properties and their ability to transfer regulatory molecules, investigators are developing several strategies for disease treatment via exosomes. These approaches will be discussed in subsequent sections. Notwithstanding, some important issues with exosome-based therapies still need to be overcome. They include difficulties in obtaining samples with the required cargo and questions on any long-term post-therapy effects. The potential advantages and disadvantages of exosome-based therapies for cardiac fibrosis are presented in Figure 3.

In order for exosome-based therapies to become realistic candidates for adoption as the treatment of cardiac fibrosis in the clinical practice, we propose that they should satisfy at least some of the following six aspirations, thus being able to (i) limit fibrosis through reduction of collagen deposition in the myocardium and/or inhibition of pro-fibrotic factors; (ii) reduce formation of myofibroblasts in the heart; (iii) be cardioprotective, i.e., reduce apoptosis of CMs and other cell types; (iv) promote blood flow recovery by increasing microvascular density; (v) selectively target cells involved in the disease with a therapeutic molecular cargo personalized to each cell type; (vi) improve cardiac function. It is not yet possible to satisfy all these ambitions due to limitations in our mechanistic understanding of the exosome properties, which is partly linked with the need to perfect the technologies available for exosome preparation as naïve. Outstanding issues include contamination with endogenous material, high variability in exosome composition, and a lack of clarity in the long term effects of exosomes. Additionally, the technologies for exosome engineering at membrane (to improve selective cell type targeting) and cargo level are still to be perfected. The successful production of therapeutic exosomes depends upon the resolution of a series of technical hurdles. At the same time, acquiring a deeper understanding of mechanisms and potential targets of cardiac fibrosis is crucial for treatment design. A potential key advantage of using exosomes therapeutically is that these vesicles, once properly harnessed, are able to improve the transfer of biological and synthetic therapeutics into targeted recipient cells, including proteins and noncoding RNA, and also deliver viral vectors for gene therapy and synthetic drugs.

### 2.1. Potential of ncRNA Targeting for the Treatment of Cardiac Fibrosis

#### 2.1.1. microRNAs

miRNAs are small endogenous oligonucleotides of 21–25 nucleotides, which are found in both animals and plants. miRNAs play an important role in post-transcriptional gene regulation by binding and generally inhibiting target mRNAs [85]. The interaction with the mRNA is usually mediated by a “seed” sequence near the 5′-terminus of the miRNA. The “seed” sequence consists of 6–8 nucleotides and can be highly conserved among species. Since the regulation of miRNAs is dependent upon sequence, the complementary principle indicates that one mRNA can be silenced by several miRNAs and one miRNA can target various mRNAs. There are a number of miRNAs which are produced by the cardiac cell types which could contribute to or alleviate a variety of pathologies, including cardiac fibrosis [86,87].

Different loading techniques can be considered for increasing the level of therapeutic nucleic acids in exosomes. Approaches already adopted include electroporation [88,89] and calcium chloride-mediated transfection [90]. Alternatively, miRNAs of interest can be loaded into exosomes by overexpression with lentiviral self-inactivating constructs in mesenchymal stem cells (MSCs). The latter approach was tested in vitro and in vivo to treat renal fibrosis targeting TGF-β/Smad cascade with let7-c (miRNA). Indirect co-culture of modified MSC with rat kidney tubular epithelial cells revealed that let-c derived from exosomes was able to decrease collagen type IVa1 and α-SMA gene expression in the kidney cells. Moreover, the let-7c-enriched exosomes proved capable of fibrosis reduction and improved structural repair of in a mouse model of kidney damage, caused by ureteral obstruction [91]. Inhibitors of profibrotic miRNAs can also be enriched into EVs through manipulation of parent cells and used as a therapy [92]. It is known, that miR-19a, miR-22, miR-132, miR-144, miR-146a, miR-181b, miR-210, miR-221, and miR-294 derived from exosomes exhibit the antifibrotic characteristic [93,94,95,96,97,98,99]. Additional antifibrotic could be considered for exosome-based treatments. Unfortunately, a lack of selectivity and the insufficient bio-distribution remain important issues. In this respect, we cannot exclude that miRNAs with a desirable therapeutic effect in cardiovascular cells, may also have detrimental effects in other cells (e.g., tumorigenesis).

Exosomes contain many miRNAs and the exosomal miRNA cargos vary in exosomes from different cell types and in responses to diseases. In order to understand which miRNAs derived from exosomes can be useful or detrimental in a treatment, the distinct role of each miRNA in pathological cardiac remodeling must be identified. Simply grouped, miRNAs associated with cardiac fibrosis can be broadly divided into anti-inflammatory/fibrotic, non/pro-inflammatory but anti-fibrotic miRNAs, non/anti-inflammatory but pro-fibrotic miRNAs, pro-inflammatory/fibrotic miRNAs, and cardioprotective miRNAs.

A miRNA with a controversial role in cardiac fibrosis is miR-21. In the work of Song et al. [92], miR-21 was overexpressed in a human embryonic kidney cell line (HEKT293T); the EVs isolated from these cultures displayed high miR-21 levels. In vitro, miR-21-enriched EVs decreased apoptosis of CMs and endothelial cells (ECs) due to PDCD4 downregulation. Moreover, EVs positive for miR-21 injected directly into infarcted area of MI mice led to a reduction in relative scar and to improved cardiac function after 4 weeks [92]. These results correlate with the recent work of Qiao and colleagues [100], who showed that dysregulation of miR-21-5p in exosomes derived from heart failure patients, reduced the regenerative potential of the heart. The authors suggested that miR-21-5p promotes angiogenesis and decreases CM apoptosis targeting the Pten/Akt pathway in vitro [100]. Wang et al. [101] showed that EVs derived from induced pluripotent cells (iPSCs) and enriched with miR-21-5p resulted in reduction of CM apoptosis through suppression of the pro-apoptotic Caspase 3. Moreover, cardioprotective properties of these vesicles were identified: pretreatment with exosomes derived from iPSCs significantly reduced CM apoptosis after myocardial ischemia/reperfusion injury in mice [101]. Additional studies showed that miR-21-5p promotes cardiac fibrosis targeting Smad, MAP kinase, and Notch1 pathways [102,103,104,105], indicating that miR-21-5p action may be highly context dependent. Most importantly, there is concerning evidence of a role of miR-21-5p in cancer [106,107]. Although these results are convincing, other elements should be also considered. One of them is the role of miR-21-3p produced by the 3′ arm of the miR-21 hairpin. Bang et al. [102] showed that cardiac fibroblast-derived exosomes contained a relatively high abundance of many miRNA passenger strands, including miR-21-3p. Exosomal miR-21-3p from angiotensin II (Ang II)-stimulated fibroblast induced cardiomyocyte hypertrophy in vitro, at least in part, by inhibiting SORBS2 or PDLIM5. Accordingly, silencing of these targets in cardiomyocytes induced hypertrophy. In mice, inhibition of miR-21-3p reduced progression of cardiac hypertrophy induced by chronic Ang II infusion [102].

Exosomes enriched with certain miRNAs can boost or attenuate scar formation, cardiac fibrosis, and inflammation by regulating gene expression. Exosomes containing miR-24-3p derived from MSCs can preserve myocardial function after MI, reduce fibrosis and inflammation, inhibit cardiac fibroblasts transdifferentiation, promote proliferation in CMs, and decrease apoptosis [108]. Antiapoptotic effects have also been observed in exosomes enriched with the miRNA-24 derived from rat plasma after remote ischemic preconditioning [109]. The ability to decrease scar formation, myofibroblast proliferation, infarct zone size and inflammation was also identified in exosomes containing miR-132 and miR-221 [95,110,111,112]. At the same time, some exosomal miRNAs were shown to have anti-fibrotic properties only, such as miR-22, miR-29, miR-29b, miR-294, miR-378, and miR-455. Evidence on the role of miR-181b is contradictory, the miRNA has been reported to reduce scar size and have cardioprotective properties whist others indicate that miR-181b derived from macrophages can promote inflammation [94,95,108,113,114]. Conversely, some miRNAs can promote cardiac fibroblasts proliferation and transdifferentiation with further scar progression. Exosomal miR-34, miR-27a, and miR-28a, derived from cardiac fibroblasts in a rat HF model, contributed to dysregulation of the Nrf2/ARE signaling pathway and led to myocardial dysfunction and heart inflammation [108,115,116]. Moreover, miR-130a, miR-208a, and miR-328 derived from exosomes were found to induce and enhance cardiac fibrosis [117,118,119,120]. Finally, there are some exosomal miRNAs like miR-210 which are considered as anti-inflammatory when miR-146a and miR-155 were found to be pro-inflammatory [95,121,122,123,124]. All ncRNAs and their role in cardiac fibrosis and inflammation are presented in Table 1.

#### 2.1.2. Long Non-Coding RNAs

Long non-coding RNAs (lncRNAs) are a class of noncoding RNA molecules which are longer than 200 nucleotides. While their existence has been known for decades, their importance and pervasiveness has been discovered as a result of the development of high throughput sequencing technologies and the completion of large human genome sequencing projects [124]. Evidence is accumulating to suggest that lncRNAs have a function to induce and regulate cardiovascular diseases (CVDs), pathological remodeling, and other disorders [125,126]. This class of RNA contributes to various processes: epigenetic regulation, transcriptional and post-transcriptional regulation, RNA splicing and editing, despite their large size, it was reported that some lncRNAs can be transported by EVs where they are protected from degradation by RNases [127,128]. Depending upon the impact of lncRNA upon cardiac fibrosis, there are two theoretical approaches which could be developed. Pro-fibrotic lncRNAs could be inhibited. Alternatively, cardioprotective and anti-fibrotic lncRNA could be delivered by exosomes to the desired site with specificity [129].

The lncRNA NONMMUT022555 has been suggested to regulate cardiac fibrosis in a MI mouse model. While knockdown of this lncRNA displayed decreased interstitial fibrosis and improved ejection fraction, overexpression of the lncRNA promoted proliferation and differentiation of CFs through regulation of let-7d. Accordingly, overexpression of let-7d precursor prevented cardiac fibrosis and improved cardiac function with pretreatment of MI mice [130]. Another study reported that the lncRNA SRA1 promotes myofibroblast proliferation after Ang-II treatment with a mechanism acting through negative regulation of miR-148b [131]. This result candidate anti-SRA1 therapies for exosome-based therapies in CVD.

In this scenario, exosomes could be utilized as carriers of siRNAs or antisense nucleotides in order to silence target lncRNAs or to deliver a ‘top-up’ of negatively regulated miRNAs to restore the balance for transcriptional regulation in nucleus. Moreover, there are a vast number of pro-fibrotic lncRNAs such as Chaer, Meg3, MIAT, MALAT1, Wisper, and H19 (which is also known to be pro-inflammatory) [125,132,133,134,135] whose activity can be reduced by antisense oligonucleotides (ASO). The most promising class of ASOs are GapmeRs due to their ability to enter into the nucleus, however there are potentially issues with hepatotoxicity [136]. For this reason, encapsulation of these ASOs in EVs engineered for high selectivity and bio-distribution may provide a solution for treatment through lncRNA mechanisms.

In contrast to the lncRNAs which promote cardiac fibrosis, Mhrt have displayed protection of CMs from hypertrophy through chromatin remodeling [137]. All ncRNAs with their roles in cardiac fibrosis are presented in Table 1. The approach requiring the overexpression of lncRNAs in the myocardium is even more challenging due to the efficiency of lncRNA upregulation itself. In addition, depending upon the disease pathology, transient or consistent overexpression may be required. Any clinical treatment involving lncRNAs, should, like other treatments suggested in this review, have an organ and even cellular specificity to reduce unwanted side effects as is likely, due to the variability in function of lncRNAs in different cell types and tissues.

#### 2.1.3. circRNAs

Circular RNAs (circRNAs) are class of non-coding RNAs, which are produced during backsplicing of exons or from lariat introns (RR) [138,139]. In contrast with linear RNAs the 3′ and 5′ ends of a strand are linked together by covalent bonds creating a stable and conserved circular structure. CircRNAs are mostly cytoplasmic, but they have been identified also in the nucleus, as well as in EVs. A variety of mechanisms of action have been identified, such as sequestration of specific proteins, transcriptional modulation, the interference with splicing, and even translation of small proteins. An additional mechanism of action is via the sequestration of specific miRNAs, leading to the de-repression of the relevant target mRNAs.

Due to the importance of transcriptional and post-transcriptional regulation in the development of cardiac fibrosis, circRNAs may be crucial for disease progression and treatment. circRNA_000203 was found to be upregulated in the diabetic mouse myocardium, as well as in Ang-II stimulated cardiac fibroblasts. Moreover, overexpression of this circRNA in mouse CFs increased the expression of ECM proteins. circRNA_000203 might work by sponging miR-26b-5p and de-repressing its targets Col1a2 and CTGF. Accordingly, circRNA_000203 prevents the anti-fibrosis effect of miR-26b-5p in cardiac fibroblasts [140]. Another study reports that circActa2 influences the expression of α-SMA by repression of miR-548f-5p [141,142]. All ncRNAs with their roles in cardiac fibrosis are presented in Table 1.

Interestingly, Li et al.; through RNA sequence analysis, found that exosomal circRNAs are more abundant and stable than those not encapsulated in exosomes [143]. Since EV-associated-circRNAs have been discovered recently, the role of exosomal circRNAs in the development of cardiac fibrosis is currently a poorly understood topic. Any potential for a therapeutic application is complicated by evidence of a role of exosomal circRNAs in tumor progression, proliferation, and metastasis, thus highlighting the need of a detailed understanding of the relevant biological phenomena [144].

### 2.2. Protein Transported by Exosomes

EVs can transfer functional proteins, which can induce and enhance cardiac remodeling or can have cardioprotective properties and anti-fibrotic effects [145]. In a recent study by Edyta Dzialo et al.; it was shown that primary human cardiac fibroblasts induced with WNT3a and WNT5a were able to generate exosomes which contain at least partially, WNT3a and WNT5a. EVs enriched with WNT3a were able to trigger WNT/β-catenin signaling in other cardiac fibroblasts, inhibiting GSK3β activation. According to the authors, this led to a translocation of β-catenin and further myocardial fibrogenesis in mice while other work reported that loss of β-catenin in cardiac fibroblasts attenuated interstitial fibrosis induced by pressure overload [146,147]. Conversely, WNT5a enriched EVs activated the non-canonical WNT pathway, going on to activate the ERK1/2 and JNK pathways leading to the generation of the pro-fibrotic factor IL6. Both in vitro and in vivo studies have shown that heat shock protein 72 (HSP72) can bind TLR2 (toll like receptor 2, a cell surface protein) and increase STAT3 phosphorylation, consequently inhibiting cardiac fibrosis and pathological remodeling. However, HSP72 therapy with EVs has not yet been achieved. One more mechanism which indirectly participates in myocardial remodeling after MI is a release of tumor necrosis factor α (TNF-α) which leads to CM apoptosis and inflammation. Yu et al. showed that TNF-α can be transported by exosomes in CMs [148]. Suppression of such pro-fibrotic exosomes can improve cells survivability and improve recovery after MI. An alternative strategy based upon exosomes encapsulated with clusterin could be employed. Clusterin is a heterodimeric glycoprotein generated almost in all human tissues. Clusterin addition to the pericardial sac in mice 7 days post-MI resulted in improved arteriolar length, density, and cell viability [149]. Thus, injections of EVs encapsulating clusterin could be cardioprotective.

Endogenous proteins within exosomes tend to promote CM apoptosis, myofibroblast proliferation, and differentiation. Inhibiting these mechanisms, signaling pathways, and functional molecules which underlie pro-fibrotic vesicle formation could be a therapeutic approach in the future. Caution must be exercised however, as manipulation of the molecular mechanisms associated with cell proliferation and inhibition could reduce cell viability. Proteins are also presented in Table 1.

### 2.3. Stem Cell-Derived Exosomes

Animal studies with stem cells have revealed an opportunity to recover CM loss after MI and improve cardiac function. Despite the advantages of stem cells, there are various negative side effects and obstacles, such as low engraftment, autoimmune response, and potential oncogenesis that limit clinical approaches [150]. Fortunately, exosomes released by stem cells have similar cardioprotective effects without the negatives of the cell therapies [151].

Exosomes derived from embryonic stem cells (ESCs) and injected into an MI mouse were shown to improve cardiac function. Moreover, EV injection led to a reduction in CM apoptosis and fibrosis. miR-294 was found to be responsible for the positive effects [152]. Crucially, it has been identified that miR-294 is not expressed in humans; a search for human analogues may resolve this problem. A further negative aspect of therapies based upon EVs from human ESCs are the ethical issues preventing the full potential of ESC work in several countries. Human iPSCs have been proposed as an ethical acceptable alternative to ESC can be generated by transfection of adult cells with a cocktail of specific genes. It was reported that paracrine regulation through exosomes is a basis of iPSCs-CMs effect on apoptosis, inflammation, fibrosis, pathological remodeling, and necrosis reduction [153]. A significant decrease in cell apoptosis and cardiac fibrosis, as well as in cardiac function improvement, was also observed in a model of dilated cardiomyopathy triggered by doxorubicin by Vandergriff et al. after the injection of exosomes derived from cardiac stem cells [154]. The positive influence of exosomes derived from stem cells depends upon their cargo, particularly miRNAs. It was identified that MSCs and exosomes collected from them have a similar expression pattern of miRNAs. miRNAs involved in cardiac fibrosis were described in the work of Shao et al. and discussed in the miRNAs chapter [108]. Feng and colleagues found that exosomes derived from bone marrow-derived MSCs with ischemic preconditioning contain large quantities of miR-22. Further in vivo studies showed reduced infarct size and cardiac fibrosis in a post-MI mouse model [94]. Another important miRNA for cardioprotection was reported in the research of Bin Yu and colleagues who showed how EVs derived from MSCs which were pretreated with GATA4 performed cardioprotective effects, induced angiogenesis, and displayed anti-apoptotic properties. Overexpression of GATA-4 in MSCs causes intensive growth factor release and regulation of miR-expression. MiRNA-19a was found to be the most regulated in comparison with a control group of EVs derived from non-treated MSCs. Treatment with these vesicles leads to attenuation of PTEN and further activation of Act and ERK in CMs, which improves cells survivability and increases proliferation [93].

In summary, stem cell-derived EVs enriched with anti-fibrotic functional molecules display promising properties for the treatment of cardiac fibrosis. Furthermore, exosomes protect precious functional cargo derived from stem cells from degradation, autophagy, and undesirable random uptake by unwanted cell types. However, for the purpose of commencing clinical applications, isolated vesicles should be isolated with a high purity and, unfortunately, most of the currently utilized techniques struggle to produce high quality vesicles. Additionally, a large number of bioactive molecules in EVs can lead to negative drawbacks, such as tumorgenesis. Thus, improvements in the techniques for isolation of exosomes and other vesicles and for proper control of EVs cargo are required.

### 2.4. Exosome-Based Therapy: Is It Specific?

Specificity is an important problem that any treatment should look to overcome, this is also true for any EV therapy. Exosomes and other vesicles should preferentially target cells involved in or contributing to the pathological remodeling associated with cardiac fibrosis. Even though some publications identify that efficiency and mechanism of exosome uptake depends on the cell type, size of the vesicles, and their surface proteins, the authors themselves admit the fact that EV subtypes usually share common surface proteins and that, whether the particular combination of EVs subtype and acceptor cell corresponds to cells specificity or this process, is unspecific [155,156,157]. Nevertheless, strategies for synthetic and natural cargo delivery with EVs are developing and two main approaches can be distinguished. The first approach is focusing on direct manipulations of vesicles and their membranes and the other is based on cellular modifications of exosome biogenesis, gene and protein expression. Both approaches will be discussed below.

### 2.5. Direct Exosome Engineering for the Treatment of Cardiac Fibrosis

Direct encapsulation of functional cargo or drug molecules can be an effective therapeutic approach to protect cells from drug toxicity and endogenous material from degradation. Curcumin (bioactive component of turmeric) has been documented as a potent anti-inflammatory reagent which affects the activity of several transcriptional factors such as NF-κB, STAT3, Nrf2, and TGF-β1 [158,159,160,161]. Jin Ma et al. showed that curcumin decreased interstitial and perivascular myocardial collagen deposition [161]. Certain successes were achieved by Sun and colleagues in the encapsulation of curcumin into EL-4 (mouse lymphoma cell line) derived vesicles. Since curcumin is a hydrophobic drug, a protocol based on sucrose gradient ultracentrifugation resulted in the hydrophobic tails of the drug becoming trapped in exosomes. Not only does encapsulation protect curcumin in the blood stream, but it also improves drug solubility, stability, and bioavailability [158]. Consequently, encapsulated curcumin may have the potential to be effectively used in treatment of cardiac fibrosis. However, the protocol suggested by Sun and colleagues can only be used for hydrophobic drugs and inhibitors, meaning it limits the number of compounds available to be used to inhibit the main drivers of cardiac fibrosis. In order to address this, more active encapsulation techniques can be applied. Active drug insertion into exosomes has been developed and described by Matthew J., Haney and colleagues for Parkinson’s disease therapy [162]. Investigators compared several active cargo-loading techniques such as loading of catalase along with (i) incubation with and without saponin, (ii) freeze-thaw cycles, (iii) sonication, and (iv) extrusion was analyzed and compared by investigators. The study found that sonication of EVs and drug with catalase leads to higher loading efficiency without significant alterations in exosome structure and sustained release of enzymatically active catalase. Nevertheless, all techniques listed above (except incubation) require exosomal bilayer disruption and further loading of cargo inside exosomes. As an alternative approach, some groups are developing EV-imitating structures. Engineering of EV-mimetics have a list of potential advantages such as enhanced targeting, as well as controllable and scalable settings [163]. The most promising competitor for EVs in drugs delivery are liposomes [164], which can transport nucleic acids, proteins, and drugs. Moreover, liposomal transport has already been used in clinical trials for breast cancer therapy where the structures were able to deliver doxorubicin to cancer cells [165,166]. In the context of cardiac fibrosis, liposomes have shown promising results [167]. The authors induced myocardial infarction in mice by permanently occluding the left main coronary artery with an intramural suture. Injection of a solution containing liposomes intro the myocardium (infarct zone) consequently had an anti-inflammatory effect and increased angiogenesis. Currently, it seems that direct engineering of exosomes has a wide spectrum of opportunities for the treatment of many diseases including cardiac fibrosis. Modification of exosomal membrane potentially can increase selectivity and bio-distribution of vesicles and various loading techniques allows choice in approaches for the targeting of specific fibrotic pathways.

### 2.6. Indirect Exosome Engineering in the Treatment of Cardiac Fibrosis

Another approach of EV engineering comes from a different angle; instead of modifying exosomes and other vesicles, parent cells are manipulated in order to produce vesicles with the necessary alterations to increase their targeting ability [168]. A technique based on the transfer of gene encoding for the exosomes targeting proteins into parent cells, has been proposed [169]. Based on the protocol of the Alvarez-Erviti L group, Kyle I., Mentkowski et al. have optimized the process for in vitro for mice-derived CMs. Since the biodistribution of cardiosphere cell (CDC) derived exosomes demonstrated low accumulation in heart, the authors fused cardiomyocyte-specific binding peptide to the extra-exosomal N-terminus of murine transmembrane protein Lamp2b in order to improve cardiac tropism of vesicles; this had no effect upon the exosome morphology. Furthermore, it was clearly shown that uptake of exosomes by CMs was enhanced and cardiomyocyte apoptosis significantly decreased. Lastly, bio-distribution was improved, and modified exosomes were observed to accumulate in the heart [170]. The same approach with other myocardium targeting peptides was used in independent studies [171,172]. It was shown that constructed Lamp2b with IMTP plasmid integrated in exosomes accumulates more in hypoxia-injured H9C2 cells than blank exosomes. Unfortunately, the antiapoptotic effect of designed vesicles was not statistically significant. However, ex vivo studies on bio-distribution in a mouse MI model studied with 1,1′-dioctadecyl-3,3,3′,3′-tetramethyl indotricarbocyanine iodide (Dir) displays significant accumulation of EVs in the area of the injury. Moreover, in vitro studies have confirmed targeting abilities of modified exosomes and their anti-inflammatory effect, as a result, expression of proinflammatory factors TNF-α, IL-6, and IL-1β was reduced dramatically. Finally, in vitro studies have shown increased vasculature, reduced myocardial apoptosis, and decreased collagen fiber length. Critically, it has been shown that treatment of mice with IMTP-exosomes improved cardiac function after only 2 weeks of treatment [171]. Vandergiff et al. compared two chemically identical peptides with an ability to target infarcted area: cardiac homing peptide (CHP) or a scramble peptide (Scr). Their in vitro study displayed increased cell viability and exosomal uptake in neonatal rat cardiomyocytes NRCMs for CHP-designed vesicles. A combination of histology and echocardiography indicated a significant reduction in fibrotic lesions and infarct area, while ejection fraction improved. It was concluded that CHP-modified exosomes boost angiogenesis and CM proliferation [172].

Another example of indirect exosome engineering is manipulation of the loading mechanism in order to selectively load cargo into the EVs. Sterzenbach and colleagues introduced a new mechanism for loading exogenous proteins in exosomes through the conserved late-domain (L-domain) pathway [173]. Another attractive tool for protein delivery by exosomes was designed by Yim et al. [174]. This approach was based on integration of a reversible protein–protein interaction module which was sensitive to a blue light and led to protein loading into exosomes. In vivo and in vitro studies displayed a dramatic increase of intracellular cargo proteins and their functions in cells which have hosted these exosomes.

**Figure 4:**
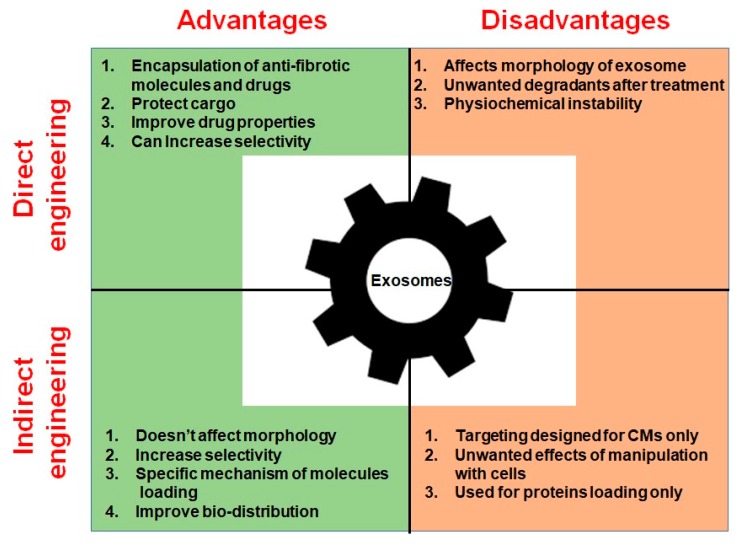
Advantages and disadvantages of direct and indirect exosome engineering.

Overall, the above studies demonstrate the vast potential of EVs as ideal candidates for the delivery of anti-fibrotic treatments. Studies of EVs modified with cardiac targeting peptides showed that the vesicles can selectively accumulate in heart and exert anti-fibrotic effects. However, possible side effects of EVs such as promotion of cancerogenesis are as yet poorly studied. The absence of a clear understanding of the cargo loading mechanism and lack of ability to obtain pure samples of certain EVs delays their use in a clinical setting [175]. Moreover, the long-term effects upon patient morbidity and mortality are not currently understood [176]. Advantages and disadvantages of both approaches are presented in Table 2 and Figure 4.

## 3. Conclusions and Future Perspectives

**Figure 5:**
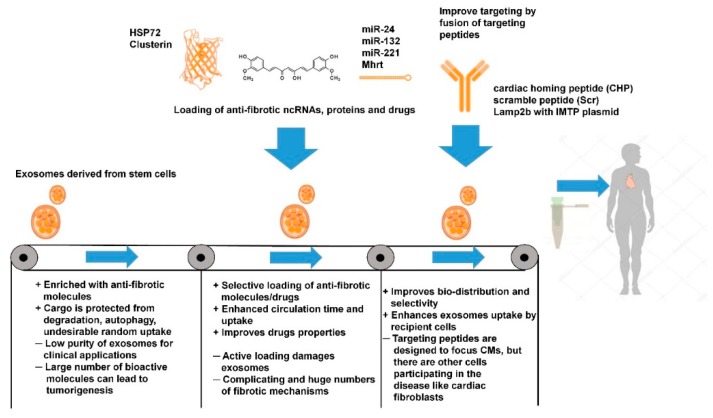
Current benefits and limitations of exosome-based therapies in the treatment of cardiac fibrosis.

In the past decade, extensive studies of EVs have provided significant new knowledge on the use, mechanisms, and cargo of these vesicles, thus providing good opportunities for the development of therapeutic strategies. Direct engineering of vesicle membranes can improve bio-distribution and selectivity (to targets). The introduction of encapsulation techniques has generated a platform for effective delivery of synthetic and biological drugs. Indirect engineering can help control exosomal cargo. Knowledge of the endogenous contents of EVs and their effect in cardiac fibrosis mechanisms allow us to consider strategies which could attenuate diseases. However, the use of EVs in a clinical environment remains challenging, primarily due to limitations in the techniques for EV isolation and characterization. The difficulties in obtaining pure fractions of exosomes with functional cargo could lead to unexpected undesirable drawbacks, including an immune response and tumor development. Secondly, usage of naturally secreted exosomes both from stem cells and cardiac cells is complicated by high variability and the diverse composition of their cargo. Finally, cardiac fibrosis is a complicated disease which includes various cell types contributing to the disease and a huge number of molecules regulating the disease at the transcriptional, post-transcriptional, and post-translational levels. Consequently, exosome-based therapies for the treatment of cardiac fibrosis, despite the many potential benefits, are currently unattainable (Figure 5). Thus, for a clear understanding of what exosomes should trigger in pathological remodeling further research is obligatory.

## Figures and Tables

**Table 1:** Endogenous cargo of exosomes which are identified to modulate inflammation or fibrosis.

**Exosomal Cargo**	**Inflammation**	**Fibrosis**	**Other Functions**	**Reference**
*miRNAs*				
miR-19a	ND	ND	Improves cell survivability Increases proliferation	[93]
miR-21-5p	ND	ND	Reduces apoptosis Improved cardiac function Cardioprotective	[92,100]
miR-22	ND	ANTI	Reduces apoptosis Reduces infarct size	[94]
miR-24	ANTI	ANTI	Preserves myocardial function after MI Inhibits cardiac FB transdifferentiation Promote proliferation of CMs Reduces apoptosis	[108,109]
miR-27a	PRO	PRO	Inhibits Nrf2 Increases oxidative stress after MI	[108,116]
miR-28a	PRO	PRO	Inhibits Nrf2 Increases oxidative stress after MI	[108]
miR-29	ND	ANTI	Reduces scar formation Reduced infarct zone Reduced MFB proliferation	[113]
miR-29b	ND	ANTI	Decreases levels of MMP9	[113]
miR-34	PRO	PRO	Reduces cardiac function Induces apoptosis	[108,115]
miR-130a	ANTI	PRO	Induces angiogenesis	[117,118]
miR-132	ANTI	ANTI	Reduces apoptosis Induces angiogenesis	[95,110]
miR-144	ND	ND	Reduces infarct size Improves cardiac function	[96]
miR-146a	PRO	ND	Reduces apoptosis Improves cardiac function	[122,123]
miR-155	PRO	ND	Increases cardiac rupture Suppresses cardiac fibroblast proliferation	[124]
miR-181b	PRO	ANTI	Reduces scar size Cardioprotective Attenuates NF-κB	[97]
miR-208a	ND	PRO	Induces FB proliferation Induces MFB activation	[119]
miR-210	ANTI	ND	Reduces apoptosis Induces angiogenesis Improves cardiac function	[95,121]
miR-221	ANTI	ANTI	Reduces apoptosis Reduces autophagy Inhibits MFB activation Reduces infarct size Improves cardiac functions	[111,112]
miR-294	ND	ANTI	Reduces CM apoptosis Cardioprotective	[95]
miR-328	ND	PRO	n/a	[120]
miR-378	ND	ANTI	n/a	[108,114]
miR-455	ND	ANTI	Decreases levels of MMP9	[113]
*lncRNAs*	**Inflammation**	**Fibrosis**	**Other Functions**	**Reference**
NONMMUT022555	ND	PRO	Reduces cardiac function Promotes FB proliferation and differentiation	[130]
SRA1	ND	PRO	Promotes FB proliferation	[131]
Chaer	ND	PRO	Causes CM hypertrophy	[125]
Meg3	ND	PRO	Regulates MMP-2 production Causes CM hypertrophy Reduces angiogenesis	[125]
MIAT	ND	PRO	Reduces cardiac function Promotes FB proliferation	[125]
H19	PRO	PRO	Increases production of ECM components (collagens, fibronectin)	[125,132]
MALAT1	PRO	PRO	Reduces cardiac function Induces FB proliferation Increases production of ECM components (collagens, α-Smooth muscle actin)	[125,133,135]
Wisper	ND	PRO	Regulates FB gene expression for cell identity, ECM, cell proliferation, and survival	[125,135]
Mhrt	ND	ANTI	Reduces cardiac hypertrophy	[125,137]
*circRNAs*	**Inflammation**	**Fibrosis**	**Other Functions**	**Reference**
circRNA_000203	ND	PRO	Prevents the anti-fibrotic effect of miR-26b-5p Promotes FB proliferation	[140]
circActa2	ND	PRO	Increases expression of α-smooth muscle actin	[141,142]
*Proteins*	**Inflammation**	**Fibrosis**	**Other Functions**	**Reference**
WNT3a	ND	PRO	Causes β-catenin accumulation and translocation	[146]
WNT5a	ND	PRO	Causes release of IL-6	[146]
HSP72	PRO	ANTI	n/a	[147]
TNF-α	PRO	PRO	Induces CM apoptosis	[148]
Clusterin	ND	ANTI	Reduces apoptosis Reduces CM hypertrophy Improves cardiac function	[149]

“ANTI” indicates anti-inflammatory/fibrotic; “PRO” indicates pro-inflammatory/fibrotic. “ND” (Not Determined) indicates that an effect has not yet been reported.

**Table 2:** Advantages and disadvantages of direct and indirect exosome engineering approaches.

Exosome Engineering	Technique	Advantages	Limitations	Reference
Direct engineering	Encapsulation of hydrophobic anti-fibrotic drugs based on sucrose gradients and ultracentrifugation	Improves drug solubility, stability, and bioavailability Protects drug in blood flow Enhances drug effect	Works only for hydrophobic drugs	[158]
Direct engineering	Encapsulation of drugs through incubation, freeze-thaw cycles, sonication, and extrusion	Allows loading of drugs and molecules inside exosomes Protects drugs from degradation	Causes disruption of exosomal bilayer	[162]
Direct engineering	Engineering of EV mimetic structures (liposomes)	Enhances targeting of drugs Increased control of structure and contents Can contain drugs and bioactive molecules	Physiochemical instability Low circulation time Can form unwanted degradants	[163,164,165,166,167]
Indirect engineering	Transfection of a gene encoding exosome-targeting proteins into parent cells	Does not affect morphology of exosomes Enhances selectivity Improves bio-distribution	Currently only CM- targeting peptides are available	[170,171,172,173]
Indirect engineering	Loading of exogenous proteins through conserved late-domain (L-domain) pathway	Specific mechanism of protein loading into exosomes Functional delivery of proteins to recipient cells	Displays only protein loading	[170]
Indirect engineering	Protein loading in exosomes based on light sensitive reversible protein–protein interaction module	Specific mechanism of protein loading into exosomes Functional delivery of proteins to recipient cells Controllable mechanism of loading	Displays only protein loading	[174]
